# Short-term elevations in glucocorticoids do not alter telomere lengths: A systematic review and meta-analysis of non-primate vertebrate studies

**DOI:** 10.1371/journal.pone.0257370

**Published:** 2021-10-01

**Authors:** Lauren Zane, David C. Ensminger, José Pablo Vázquez-Medina

**Affiliations:** 1 Department of Integrative Biology, University of California, Berkeley, CA, United States of America; 2 Department of Biological Sciences, San Jose State University, San Jose, CA, United States of America; University of Newcastle, UNITED KINGDOM

## Abstract

**Background:**

The neuroendocrine stress response allows vertebrates to cope with stressors via the activation of the Hypothalamic-Pituitary-Adrenal (HPA) axis, which ultimately results in the secretion of glucocorticoids (GCs). Glucocorticoids have pleiotropic effects on behavior and physiology, and might influence telomere length dynamics. During a stress event, GCs mobilize energy towards survival mechanisms rather than to telomere maintenance. Additionally, reactive oxygen species produced in response to increased GC levels can damage telomeres, also leading to telomere shortening. In our systematic review and meta-analysis, we tested whether GC levels impact telomere length and if this relationship differs among time frame, life history stage, or stressor type. We hypothesized that elevated GC levels are linked to a decrease in telomere length.

**Methods:**

We conducted a literature search for studies investigating the relationship between telomere length and GCs in non-human vertebrates using four search engines: Web of Science, Google Scholar, Pubmed and Scopus, last searched on September 27th, 2020. This review identified 31 studies examining the relationship between GCs and telomere length. We pooled the data using Fisher’s Z for 15 of these studies. All quantitative studies underwent a risk of bias assessment. This systematic review study was registered in the Open Science Framework Registry (https://osf.io/rqve6).

**Results:**

The pooled effect size from fifteen studies and 1066 study organisms shows no relationship between GCs and telomere length (Fisher’s Z = 0.1042, 95% CI = 0.0235; 0.1836). Our meta-analysis synthesizes results from 15 different taxa from the mammalian, avian, amphibian groups. While these results support some previous findings, other studies have found a direct relationship between GCs and telomere dynamics, suggesting underlying mechanisms or concepts that were not taken into account in our analysis. The risk of bias assessment revealed an overall low risk of bias with occasional instances of bias from missing outcome data or bias in the reported result.

**Conclusion:**

We highlight the need for more targeted experiments to understand how conditions, such as experimental timeframes, stressor(s), and stressor magnitudes can drive a relationship between the neuroendocrine stress response and telomere length.

## Introduction

The vertebrate neuroendocrine stress response integrates external stimuli into a broad range of physiological adjustments through the activation of the Hypothalamic-Pituitary-Adrenal axis (HPA axis) and the concomitant secretion of glucocorticoids (GCs) [1, 2]. While the primary GC produced varies by taxa (e.g., cortisol in humans and corticosterone in birds and other mammals [3]), the impacts of GCs on organismal physiology are remarkably similar. Across species, an increase in GC secretion can typically be detected in 3–5 minutes following interaction with a stressor [4]. Additionally, GCs are relatively easy to quantify because they are present in all vertebrates and can be measured noninvasively in multiple matrices including hair and feces using a variety of assays [5, 6]. Therefore, wildlife stress physiology studies often rely on GC measurements as an indicator of the neuroendocrine stress response [7]. Following their secretion, GCs induce a myriad of acute behavioral and physiological effects to prioritize immediate survival [8, 9].

In addition to allowing animals to cope with immediate stressors, GCs can influence other cellular processes such as telomere length dynamics. Telomeres are evolutionarily conserved caps that protect chromosomes against the loss of coding nucleotides during cell replication and against chromosomal fusion [10]. Telomere shortening is associated with aging, the neuroendocrine stress response, and survival, and is thus of interest to several fields of biology [1, 11]. In humans, increased telomere loss predicts the onset of age-related diseases, cardiovascular complications, cellular senescence, and other aging phenotypes [12, 13]. Telomere attrition can be attributed to several causes including the “end replication problem” in which the terminal end of linear DNA cannot be completely replicated by the lagging strand [14]. Since the end replication problem occurs at every cell division, telomeres continuously shorten with age progression [15]. Other stressors such as inflammatory challenges erode telomeres regardless of age [16].

In non-human vertebrates including birds, mammals, fish, amphibians and reptiles, exposure to challenging environmental conditions correlates with shorter telomeres [17, 18]. Reproductive stressors such as an artificially increased brood size can also shorten telomeres in zebra finch parents compared to controls and parents with a reduced brood size [19]. Early telomere length is positively correlated with survival and lifetime breeding success in both wild purple-crowned fairy wrens and zebra finches. Thus, individuals with longer telomeres are more likely to survive and produce more offspring that survive to maturity [20, 21]. Therefore, telomere dynamics—the change in telomere length attributed to processes of elongation and shortening—is related to organismal fitness [22]. In addition to impacting telomere length, stressors that lead to energy limitation such as psychological stress, disease, accelerated growth, nutrient shortage and work load activate the HPA axis causing the release of GCs [23].

Thus, several hypothesized connections between GCs and telomere length exist. Firstly, GCs are an essential part of the vertebrate stress response, and their primary function is to mobilize energy [5]. Accordingly, the “metabolic telomere attrition hypothesis” proposes that during events that require an increased amount of energy and metabolic rates, telomeres are shortened as collateral [20]. As a result of the high energy expenditure, the energetically expensive maintenance of telomeres cannot take place as an emergency survival mechanism due to a shift in energy allocation [23]. In addition, GCs stimulate the generation of reactive oxygen species (ROS) and subsequent oxidative damage to telomeres, which are particularly susceptible to oxidation due to a high guanine content [11, 24–26]. Finally, cortisol reduces telomerase—the enzyme responsible for telomere maintenance—activity in human T lymphocytes [27]. This reduction in telomerase activity can result in excessive telomere attrition [28]. Since wildlife face an array of stressors throughout their lifetime and these stressors can erode telomeres, GCs may play a mechanistic role in telomere loss [1].

External stressors cause pleiotropic effects that can potentially influence telomere dynamics, however the evidence for a causal relationship between GCs and telomere length is sparse. Two recent literature reviews on the topic by Angelier *et*.*al* 2018 and Casagrande and Hau 2019 [11, 23] summarize the potential relationship between GCs and telomere length. However, it is essential to build a quantitative understanding of the relationships between the neuroendocrine stress response and its downstream effects. In this study, we review the existing literature for empirical evidence of the relationship between GC secretion and telomere length to better understand the underlying mechanism of telomere shortening as well as potential consequences of the neuroendocrine stress response in non-primate vertebrates. Using a meta-analytical framework, we tested whether GC levels impact telomere length and if this relationship can differ among time frame, life history stage, or stressor type. We hypothesized that elevated GC levels are linked to a decrease in telomere length.

## Methods

### Literature search and study selection

We conducted a literature search for studies investigating the relationship between telomere length and GC levels in non-human vertebrates using four search engines: Web of Science, Google Scholar, Pubmed and Scopus. Five subsets of the following keywords ‘reactive oxygen species,’ ‘antioxidant,’ ‘glucocorticoid,’ ‘cortisol,’ ‘corticosterone,’ ‘telomere length,’ ‘chronic stress,’ ‘oxidative stress,’ ‘acute stress,’ ‘chronic stress,’ ‘telomeres,’ and ‘HPA axis,’ were conducted in each search engine. We did not specify a time frame in our literature search. Additional records were obtained from the reference section of studies included in the meta-analysis. Our study includes a qualitative synthesis of 31 full-text, peer-reviewed studies, and we report effect sizes for 15 of these studies.

Studies were excluded if (1) GCs were administered, but physiological measurements such as feather or plasma GC levels were not taken. Such studies were excluded because it would not be possible to calculate the appropriate effect size (Fisher’s Z) for correlation data. For homogeneity in effect size calculation and statistical analysis, we did not include studies in which (2) GCs and telomere length were not specifically measured at two different time points (before or after treatment) (3) raw data was not accessible to use for the effect size calculation, or (4) telomere length measurements or GC measurements were log transformed.

### Statistical data analyses

#### Meta-analysis

We conducted statistical analyses exclusively on studies with raw data available. When data was not publicly accessible, we contacted authors via email for consensual access. For each study, the correlation coefficient (R^2^) was calculated by fitting a linear mixed model using the “lme4” R package (version 3.6.1, R Development Core Team, Boston, MA). When possible, random effects such as multiple blood draws from a single individual were incorporated in the linear mixed model (LMER) to account for variability not captured by explanatory parameters. For studies where a random effect could not be determined, a linear model (LM) was fitted. From the LMs and LMERs, R^2^ values were obtained from the model and converted into Fisher’s Z, then adjusted for sample size and combined into a pooled effect size (Fisher’s Z; Z) using the R package “meta”. The random-effects model meta-analysis was implemented in our study as this model accounts for the assumption that studies come from different populations, rather than the same population. These pooled effect sizes were then visualized in a forest plot.

The “meta” package was also used to assess the statistical difference between observed and fixed effect model estimate of effect size (Cochrane’s Q) and the percent of variability in effect sizes that is not caused by sampling error (I^2^). After estimating heterogeneity, we identified potential outliers. Studies were classified as outliers if the study had an effect size with a confidence interval that did not intersect with the confidence interval of the pooled effect size.

Since some studies can have a larger influence on the pooled effect size than others due to its sample size or individual effect size, we conducted an influence analysis. The analysis was conducted by omitting each study one at a time and simulating the pooled effect size, with a confidence interval had the study not been included. This influence analysis was represented in a Baujat plot, which shows the contribution of each study to heterogeneity as Cochrane’s Q, and compares this to the study’s influence on the pooled effect size.

#### Subgroup analysis

Since experimental design can affect the outcome of a study, differences in effect size may be attributed to these variables. As such, further sources of between-study heterogeneity were investigated through subgroup analysis and meta-regression. In the subgroup analysis, studies were grouped based on different categorical experimental parameters. We completed eight different subgroup analyses for the following parameters—duration of stressor, type of GC assay, telomere assay, species, taxa, study type, life history stage, and stressor type. For each subgroup analysis, a pooled effect size (Fisher’s Z) was calculated. We then compared pooled effect sizes and tested for between-study subgroup differences. The meta-regression was analogous to the subgroup analysis, except the parameter of investigation is continuous rather than categorical. We conducted one meta-regression for publication year and subsequently tested for between-study subgroup differences. For all analyses the significance threshold was set at p<0.05.

In the subgroup analysis, studies included in the meta-analysis were clustered based on categorical grouping and represented as a pooled effect size with a 95% confidence interval. The between study difference was indicated by Cochrane’s Q and the subsequent p-value for this statistical measure. The first subgroup analysis “stressor duration” organized studies based on the timeframe of the experiment—less than one week (n = 1), one to two weeks (n = 2), two to three weeks (n = 7), three to four weeks (n = 1), or longer than four weeks (n = 4)—. The second subgroup analysis, “type of stress” compared anthropogenic (n = 5) to naturally occurring stress (n = 7), or if stress was simulated by GC administration (n = 3). The subsequent subgroup analysis “life history stage, “differentiates studies based on pre-maturate study organisms (n = 12), or post-maturate study organisms (n = 3). Next, the subgroup “GC assay,” separates studies into those that quantified plasma GCs (n = 13) or non-plasma GCs (n = 2). Similarly, by performing the subgroup analysis for the variable “telomere assay” we hoped to parse out potential differences between the three methods of telomere quantification: qPCR (n = 7), TeloTAGGG (n = 1), and Telomerase Restriction Fragment (TRF; n = 7). The fifth subgroup analysis contrasts avian (n = 12) and non-avian (n = 3) studies. To explore the relationship between individual species, we performed an additional subgroup analysis for each species included in the study. Finally, the subgroup analysis “study type” distinguished studies based on study design: cross-sectional (n = 5), repeated measures (n = 2), or within individual (n = 8) design.

#### Publication bias

Published studies may not accurately represent the total studies investigating an area of research due to selective outcome reporting, missing studies and a higher likelihood of publication of studies reporting a significant (p<0.05) result. While proving selective outcome reporting and other forms of publication biases is challenging, missing studies can be visually represented using a funnel plot. Commonly, studies with small effect sizes and small sample studies are likely to be missing, which can be depicted with funnel asymmetry or holes in the funnel plot. We created a funnel plot by graphing effect size against study precision, defined as the standard error of the effect size to visualize potential publication bias. We also report an Egger’s test, which is represented by the intercept, it’s confidence interval, and the associated p-value to determine if publication bias was statistically significant.

#### Risk of bias in included studies

We assessed studies for missing outcome-level data, measurement of the outcomes, and outcome reporting in each included study. For the missing outcome-level data domain, we considered studies that could not report values for telomere length or GCs in less than 10% of total study organisms as low risk. We designated studies that did not report these values for 10–50% of study organisms as moderate risk and studies that did not report values for over 50% of GCs or telomere length, as high risk. Secondly, we based risk of bias in the measurement of outcome on the type of GC and telomere measurement. Low risk studies utilized plasma GCs or salivary GCs because these quantifications capture elevations related to a short-term stress event within minutes. Studies that measured GCs in fecal matter received a ranking of some concern because fecal GCs typically encapsulate cumulative stress over the day rather than GCs related to a particular environmental stressor. Fecal GCs also received a ranking of some concern due to potential variations related to storage and collection times, which can affect the concentration of fecal GC metabolites in a sample [29]. We considered studies that measured GCs in feathers as high risk because feathers incorporate GCs in over a month. Additionally, we considered feather GC quantification as high risk because feather preparation and GC extraction can vary greatly [30]. Finally, for the risk of bias due to outcome reporting we denoted studies that based results off a subset of time points or measurements high risk. We denoted studies that report results based on all time points with low risk. We took these three domains into consideration when assessing overall risk of bias.

## Results

### Literature search and study selection

**Fig 1:**
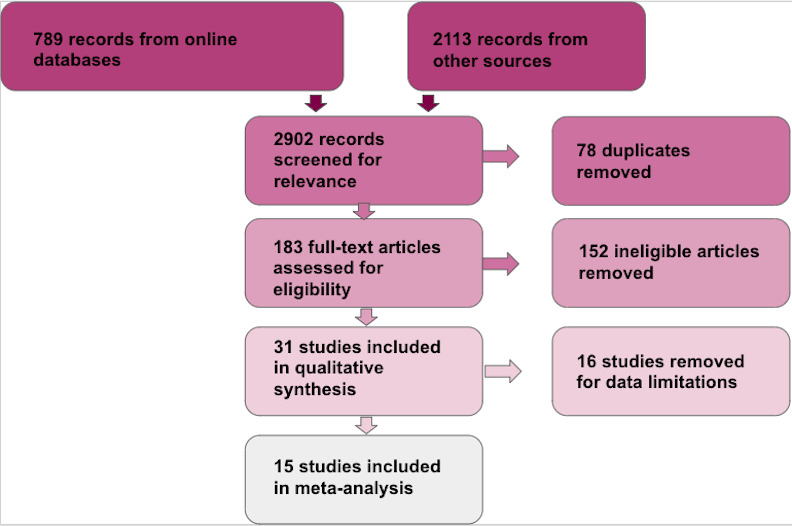
PRISMA diagram. PRISMA diagram showing the selection process for references included in the meta-analysis of the effects of GCs on telomere length.

We electronically screened 789 records for relevance from the following databases: Google Scholar (n = 512), Web of Science (n = 105), PubMed (n = 72), and Scopus (n = 100). 2113 additional records were hand screened from the reference section of the 31 studies used in qualitative analysis. Of the total 2902 records that were screened for relevance, 78 were removed as duplicates and 2,489 did not fit criteria for our study. For example, some excluded studies include human trials, cell culture work, or studies that only assessed research questions pertaining to either telomere length or GC levels, but not both (Fig 1; S1 Table). Of the 183 assessed full-text articles, we removed 152 studies that did not fulfill our inclusion criteria. We statistically analyzed 15 of the remaining 31 studies, the ones that provided raw data for analysis either within the manuscript or after contacting the corresponding author [16, 22, 29–42]. The other 16 studies appeared to fit criteria but did not provide raw data for analysis [26, 30, 43–55]. The literature and study selection process is illustrated using a PRISMA diagram (Fig 1).

### Meta-analysis

**Fig 2:**
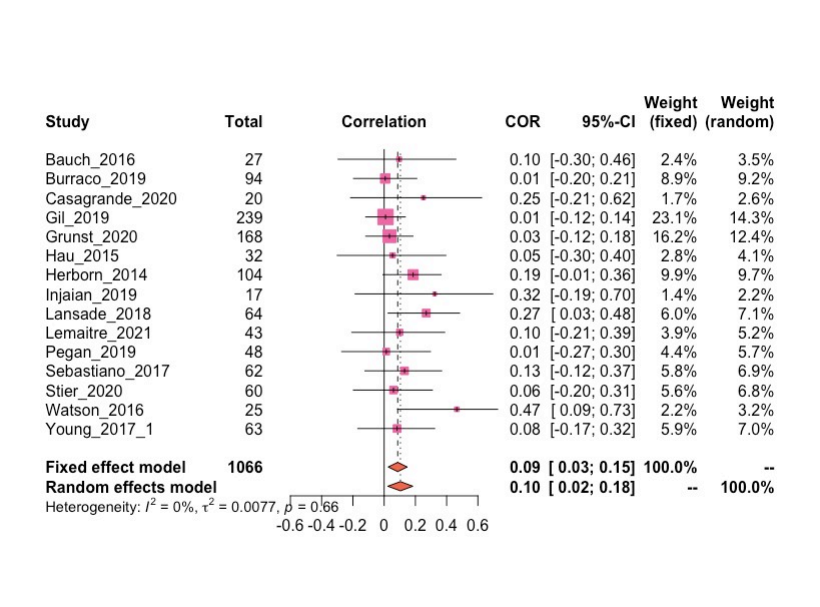
Forest plot. Distribution of effect sizes of GCs on telomere length and 95% CI of effect size. Dashed lines represent pooled effect sizes using a random and fixed effect model. Heterogeneity (I^2^), the percent of variability in effect sizes that is not caused by sampling error indicates very little variability in effect size. Weight indicates the influence the study has on the overall pooled effect.

The random-effects model meta-analysis is represented as a pooled effect size (Fisher’s Z) with 95% confidence intervals (Fig 2). No studies were removed as outliers. The model found no relationship between GC levels and telomere length (Fisher’s Z = 0.1042, CI = 0.0235; 0.1836). Both heterogeneity measures, Cochrane’s Q (Q = 11.31, p = 0.6615) and I^2^ with 95% confidence intervals (I^2^ = 0.0%; CI = 0.0%; 42.6%) yielded similar results.

The influence analysis indicated that theoretically removing one study at a time did not yield pooled effect sizes (Fisher’s Z = 0.09–0.11) that differed from the original pooled effect size (Fisher’s Z = 0.11, S1 Fig). Additionally, the influence analysis demonstrated that certain studies unevenly impacted the pooled effect size and/or overall heterogeneity (S2 Fig), but no studies were removed as outliers.

### Subgroup analysis

The subgroup analysis for “stressor duration” found no differences between any of the tested time frames (Table 1). The difference between-studies was not statistically significant (Q = 1.86, p = 0.7594). Similarly, the subgroup analysis for “stressor type,” did not reveal a difference between types of stressors (Table 1). The between study difference was not significantly different (Q = 2.56, p = 0.2783). Likewise, our subgroup “life history stage,” did not show differences between effect sizes for pre- and post-maturation organisms (Table 1), and did not indicate a difference between groups (Q = 0.06, p = 0.8119). The fourth subgroup analysis, “GC assay” did not find a difference between plasma GCs and other GC measurements, yielding a non-significant difference between studies (Q = 0.03, p = 0.8742) (Table 1). Additionally, the between study difference for the telomere assay subgroup did not find a significant difference between the three telomere quantification methods (Q = 0.12, p = 0.9401; Table 1). Our sixth subgroup analysis examined potential differences in effect size due to taxa, which could be divided into the binary categories avian and non-avian (Table 1). There was no difference between-studies (Q = 0.03, p = 0.8666). Our analysis further explored species-specific differences and accordingly did not find a significant difference between species (Q = 9.27, p = 0.6797). Similarly, the final analysis investigated potential differences between study designs and yielded a non-significant difference between cross-sectional, repeated measures, or within individual designs (Q = 1.27, p = 0.5289).

**Table 1:** Pooled effect sizes with 95% CI of experimental parameters investigated during the five subgroup analyses for stressor duration, stressor type, life history stage, GC assay and taxa group.

Experimental Parameter	Number of Studies	Effect Size	95% CI
		(Fisher’s Z)	
*Stressor Duration*			
< 1 week	n = 1	0.0135	-0.2717; 0.2965
1–2 weeks	n = 2	0.0959	-0.1663; 0.3454
2–3 weeks	n = 7	0.0741	-0.0157; 0.1628
3–4 weeks	n = 1	0.0957	-0.2950; 0.4591
> 4 weeks	n = 4	0.2181	0.0101; 0.4080
*Type of Stress*			
Anthropogenic	n = 5	0.1902	0.0059; 0.3621
Naturally occurring	n = 7	0.0451	-0.0388; 0.1284
GC administration	n = 3	0.1425	-0.0320; 0.3085
*Life History Stage*			
Pre-maturation	n = 12	0.1111	0.0185; 0.2019
Post-maturation	n = 3	0.0843	-0.1183; 0.2800
*GC Assay*			
Plasma GCs	n = 12	0.1012	0.0061; 0.1945
Non-Plasma GCs	n = 3	0.1161	-0.0437; 0.2701
*Taxa Group*			
Avian	n = 12	0.1012	0.0088; 0.1919
Non-Avian	n = 3	0.1183	-0.0600; 0.0936
Species	n = 1	0.0993	[-0.2072; 0.3881]
*Capreolus capreolus*			
*Coturnix japonica*	n = 1	0.0596	[-0.1973; 0.3089]
*Fregata magnificens*	n = 1	0.1306	[-0.1232; 0.3684]
*Hydrobates pelagicus*	n = 1	0.4651	[0.0857; 0.7267]
*Parus major*	n = 2	0.0707	[-0.1322; 0.2678]
*Phalacrocorax aristotelis*	n = 1	0.1852	[-0.0600; 0.2893]
*Rana temporaria*	n = 1	0.0067	[-0.1962; 0.2090]
*Rissa tridactyla *	n = 1	0.0826	[-0.1686; 0.3238]
*Sterna hirundo *	n = 1	0.0957	[-0.2950; 0.4591]
*Sturnus unicolor*	n = 1	0.0088	[-0.1182; 0.1356]
*Tachycineta bicolor*	n = 2	0.1134	[-0.2110; 0.4153]
*Turdus merula*	n = 1	0.0539	[-0.3004; 0.3952]
Welsh pony	n = 1	0.2693	[0.0252; 0.4831]
*Telomere Assay*	n = 7	0.1186	[-0.0089; 0.2424]
qPCR			
TeloTAGGG	n = 1	0.1306	[-0.1232; 0.3684]
TRF	n = 7	0.0909	[-0.0409; 0.2197]
*Study Type*	n = 5	0.1687	[-0.0219; 0.3474]
Cross sectional			
Repeated measure	n = 2	0.0271	[-0.1336; 0.1864]
Within individual	n = 8	0.0984	[0.0040; 0.1910]

The meta-regression was performed for the continuous variable publication year and represented as Cochrane’s Q and the associated p = value. Publication dates ranged from 2014–2021. Publication date was not a significant predictor of effect size (Q = 1.252, p = 0.2632).

### Publication bias

We found publication bias against studies with small sample size and small effect size (S3 Fig; Egger’s test for small sample bias: intercept = 1.420616, CI = 0.3753223; 2.465909, p = 0.02064949).

### Risk of bias in included studies

We represent the results of the risk of bias analysis in Table 2. Four of fifteen studies received a risk of bias ranking of moderate concern. These studies had some missing values for GCs or telomere length or selectively reported one time point in the results. The other eleven studies received a ranking of low risk and accordingly reported nearly all values for physiological parameters, measured GCs in plasma or saliva, and did not selectively report results.

**Table 2:** Overall risk of bias assessed based on missing outcome data, measure of outcome and in the selection of reported results.

Author	Year	Bias due to missing outcome data	Bias in measure of outcome	Bias in the selection of reported result	*Overall Risk of Bias*
*Bauch et*. *al*	2016	high	low	high	*some concern*
*Burraco et*. *al*	2019	low	low	low	*low risk*
*Casagrande et*. *al*	2020	high	low	low	*some concern*
*Gil et*. *al*	2019	low	low	low	*low risk*
*Grunst et*. *al*	2020	low	high	low	*low risk*
*Hau et*. *al*	2015	low	low	low	*low risk*
*Herborn et*. *al*	2014	low	low	low	*low risk*
*Injaian et*. *al*	2019	high	low	high	*high risk*
*Lansade et*. *al*	2018	low	low	low	*low risk*
*Lemaitre et*. *al*	2021	low	moderate	low	*low risk*
*Pegan et*. *al*	2019	high	low	low	*some concern*
*Sebastiano et*. *al*	2017	low	low	low	*low risk*
*Stier et*. *al*	2020	low	low	low	*low risk*
*Watson et*. *al*	2016	low	low	low	*low risk*
*Young et*. *al*	2017	moderate	low	high	*some concern*

## Discussion

External and internal stimuli can activate the neuroendocrine stress response in vertebrates, resulting in the secretion of GCs, which induces multiple downstream physiological and behavioral effects [8, 9]. GCs might directly or indirectly cause telomere erosion [1, 11, 32]. Therefore, our goal was to investigate the relationship between GCs and telomere length *via* meta-analysis using data from empirical studies. Though our sample size was limited (n = 15), our data do not support the hypothesis that elevated GC levels result in telomere shortening.

The empirical evidence for a relationship between GCs and telomere length is mixed, with some studies showing that telomere shortening is directly related to GC levels, and other studies finding no relationship. For example, GCs influence telomere dynamics in wild roe deer and great tits [32, 39], but not in red squirrels or magellanic penguins [46, 47]. These results suggest that the relationship between GCs and telomere length is species-specific. Alternatively, a potential relationship may be obscured by the methods used to measure GCs and telomere length or by differences in experimental design including time frame. A differential sensitivity of the HPA axis can also obscure conclusions made from GC measurements especially in free-ranging vertebrates that can potentially encounter a variety of external stimuli [1]. For example, since GC levels in plasma remain elevated for several minutes after a stressor subsides, it can be challenging to assess whether a measured GC increase results from the stressor in question, the stress involved in obtaining a sample from the experimental subject, or an unrelated event triggering HPA axis activation [6, 56]. As baseline plasma GC samples must be collected quickly in many species, it can be logistically difficult to attain a true baseline GC value in the field [57–60]. GCs can also be incorporated into other matrixes such as saliva, feathers, and hair [4, 58]. The multitude of non-invasive GC sampling sources is advantageous to conservation physiology as their quantification does not require capture [6]. However, across tissues and fluids, the time required for GC incorporation varies. For example, elevations in plasma GCs can be detected within minutes of stressor exposure, whereas GCs integrate into hair a week or more after stressor exposure [4]. Hence, there are caveats in the interpretation of each measurement such as incongruencies between GC levels in plasma and other tissues, hair and saliva [60]. Therefore, GC measurements in feces may be more representative of accumulated stress, rather than the event in question [6].

GC quantification in tissues and feces can also present specific uncertainty and imprecision during sampling, storage, and extraction. In fecal samples, GC metabolites can increase up to 92% in 120 days and provide an inaccurate assessment of GC levels [61, 62]. Excrement not collected immediately or across different time scales can obscure potential differences since exposure to abiotic factors like rainfall or extreme temperature can alter the concentration of fecal glucocorticoid metabolites [63]. Moreover, diet can affect GC metabolites in fecal samples, since an increased amount of cellulose depresses fecal glucocorticoid metabolite concentrations [61]. Similarly, feather preparation and extraction can also affect GC levels [64]. Further, different parts of the feather yield different concentrations of GCs. Saliva based GC extraction and quantification hosts similar shortcomings, though salivary GCs increase on a similar timeline (5–10 minutes) to circulating plasma GCs and thus prove a close proxy for plasma GC quantification [65]. Other factors such as time since last meal and recent activity also impact salivary GC measurement [66].

Similar considerations must be taken into account when assessing telomere length. Since telomere length can be influenced by environmental, maternal, and epigenetic effects, there is a large inter-individual variability in telomere dynamics [11, 67]. Several factors may contribute to this variability including discrepancies between the repeatability of different telomere measurement assays. Seven studies included in our meta-analysis utilized the telomere restriction fragment (TRF) assay, which depends on the distribution of the terminal restriction fragments to average the length of telomeres in a given cell population [68]. The other eight studies used the quantitative polymerase chain reaction (qPCR, n = 7), which relies on the quantification of the highly conserved (TTAGGG)_n_ sequence for a Southern blot variation (TeloTAGGG for telomere quantification (n = 1) [69]. TRF-based studies are highly repeatable within individuals, whereas qPCR based studies are less repeatable and more variable than TRF because they are more prone to measurement errors [70]. qPCR can also bias measurements of telomere length because some species that exhibit interstitial telomeric repeats will artificially enlarge telomere length [71, 72]. In addition to methodological differences, there is large individual variability in telomere length based on tissue type [73]. In adult zebra finches, telomere length in red blood cells is correlated with telomere length in the spleen, liver and brain, but not muscle or heart [31]. While avian studies in our meta-analysis used red blood cells for telomere measurement, telomere length was measured in tail muscle and liver in mammals and amphibians, which could lead to discrepancies when comparing among studies [31, 46, 57].

A variety of biological factors also contribute to the diversity of telomere dynamics observed within a study and the large amount of observed inter-individual variability. The rate of telomere shortening can be influenced by the life histories and environmental conditions [22]. In accordance with the metabolic telomere attrition hypothesis, shortening is exacerbated by life history stages requiring more energy, such as reproduction [32]. Within an energy intensive process like reproduction, there can be a large inter-individual variability related to reproductive effort, which can be attributed to brood size and food availability [74]. Differences in reproductive roles during the breeding season account for sex-specific telomere dynamics which can contribute to differences in the variability of telomere dynamics within a study [75]. Finally, individuals respond differently to environmental challenges which can act synergistically with rapid growth or energy intensive life stages to magnify the rate of telomere shortening in non-model vertebrates [71].

Telomere dynamics can be complicated by the presence of telomerase which in some cases can elongate telomeres [22, 76]. Typically, telomerase exhibits higher activity in developing organisms as compared to adults [77]. Ectotherms such as amphibians and reptiles have telomerase that is active throughout adulthood while endotherms reduce telomerase expression almost to non-detectable levels as they reach maturity [11, 70]. However, there is conflicting evidence on these observations, as telomerase activity has been detected in adult common terns and European Storm Petrels among other species [78, 79]. Nonetheless, adult telomere shortening is observed in chickens, which have active telomerase in the adult life stage [26]. While there is an absence of empirical evidence on the long-term activity of telomerase in many avian species, even adults exhibit general shortening trends [76].

Many factors influence GC and telomere measurements. During the subgroup analysis, we attempted to disentangle the underlying causes of the variation in effect size. Ultimately, we found no impact of stressor, taxa, type of GC assay, or life history stage on the heterogeneity of the effect size. While no subgroup was identified as a predictor of heterogeneity in effect size, pooled effect sizes in certain categories with the subgroup indicate a higher pooled effect size than the overall pooled effect size. The small sample size for some parameters precluded further statistical analysis, however, we found variables of interest that may play a large role in the relationship between GCs and telomere length. For example, within “experimental timeframe,” (n = 4) the group of studies with a timeframe above four weeks had a pooled effect size of 0.2181, while all other groups’ pooled effect size was less than that of the overall pooled effect size. Since most studies took place in less than four weeks, this suggests that while almost immediate changes in GCs can be observed, the impact of GCs on telomere length cannot be seen on short time scales. This idea is consistent with typical responses of telomere shortening observed in studies that take place for more than a year [29, 54, 79–81]. More work is needed to explore if long-term rather than short-term studies can be used to tease apart parameters that underlie the connection between GCs and telomere length such as stressor type or duration.

While GC secretion is often viewed as the endpoint of HPA axis activation in response to external stimuli, GC manipulation is an oversimplification of the stress response which involves a multitude of physiological mechanisms that can each impact energy allocation and promote telomere erosion [8]. This highlights the problematic nature of the category “GC stress” which was investigated as a category during the subgroup analysis, in which studies subjected organisms to GC manipulation *via* an implant or oral administration. Since previous research found that organismal stress can result in adverse physiological responses without the involvement of the HPA axis, these results underscore the issue of using only GCs as a proxy for stress [82, 83].

Overall, we found no relationship between GCs and telomere length across studies. Currently, the existing literature shows both a direct relationship and a lack of a relationship between GCs and telomere dynamics, suggesting that the underlying mechanisms driving this relationship are species-specific or altered by differences in experimental design. However, due to limited sample size, we are unable to investigate the underlying variables that play a role in this relationship. Here, we highlight the need for more studies with targeted experimental parameters to understand how conditions, such as experimental timeframes, stressor(s), and stressor magnitudes can drive a potential relationship between the neuroendocrine stress response and cellular aging. Thus, we recommend the following research priorities to groups studying similar questions.

1. Experimental timeframes and stressor magnitudes should be long enough to observe telomere erosion in relation to stressors when studying GCs.
2. When possible, studies should use a repeated measures design to measure cortisol levels and telomere lengths before and after stress exposure to account for individual variation.
3. While the avian taxa are well represented in this research topic, there is a dearth of information on other taxa. It will be important to investigate the neuroendocrine stress response in other vertebrates including mammals and reptiles to understand if similar principles hold true in these taxa or if telomere dynamics differ across taxa.
4. If possible, future research should assess the functionality of the study organisms’ HPA axis by ACTH/dexamethasone challenge prior to exposure to a stressor and completion of the study.

### Certainty of evidence

We utilized the applicable Cochrane/GRADE categories “risk of bias,” “inconsistency,” and “publication bias,” for the determination of the certainty of evidence. Overall, we have a moderate confidence in the certainty of evidence. While most studies received a low risk of bias assessment, and had low heterogeneity, we report a considerable amount of publication bias as evidenced by Egger’s test and an asymmetrical funnel plot.

## Supporting information

S1 Checklist PRISMA 2020 checklist. (PDF)

S1 Fig Influence analysis plot. The leave one out recalculation reveals a similar effect size across studies and indicates that studies evenly contribute to the pooled effect size.
(TIF)

S2 Fig Baujat plot. Studies can have an unequal influence on the pooled effect size and contribute to the heterogeneity of effect sizes. The horizontal axis represents Cochrane’s Q and influence on the pooled effect size on the vertical axis.
(TIF)

S3 Fig Funnel plot. The lack of studies in the bottom left of the “funnel” demonstrates publication bias against studies with small sample sizes and small effect sizes.
(TIF)

S1 Table Search strategy table. Details search term combinations used to search online databases and websites.
(XLSX)
