# Supplementary figures and images for: Short-term elevations in glucocorticoids do not alter telomere lengths: A systematic review and meta-analysis of non-primate vertebrate studies
Source: PLoS One. 2021 Oct 1;16(10):e0257370. doi: 10.1371/journal.pone.0257370 (PMC8486123; doi:10.1371/journal.pone.0257370)

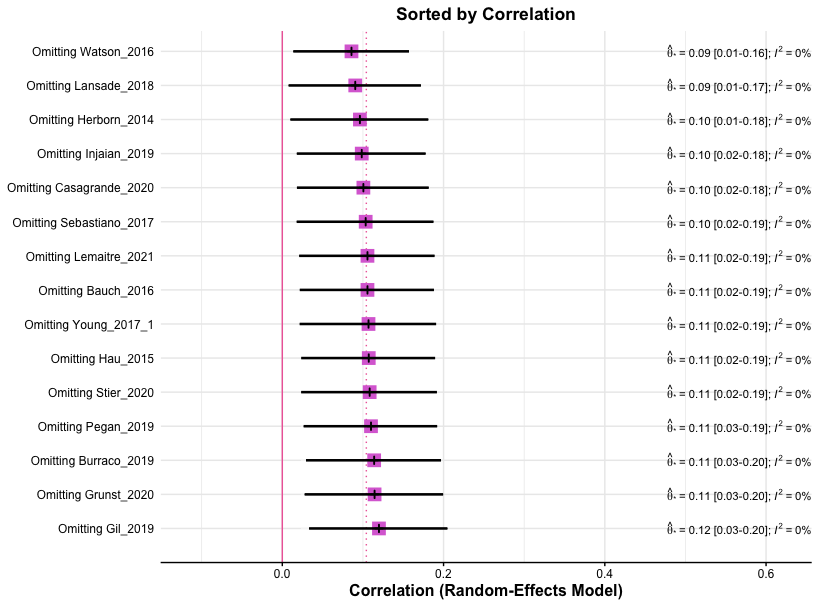

Supplement: S1 Fig — The leave one out recalculation reveals a similar effect size across studies and indicates that studies evenly contribute to the pooled effect size. (TIF) [file pone.0257370.s002.tif]

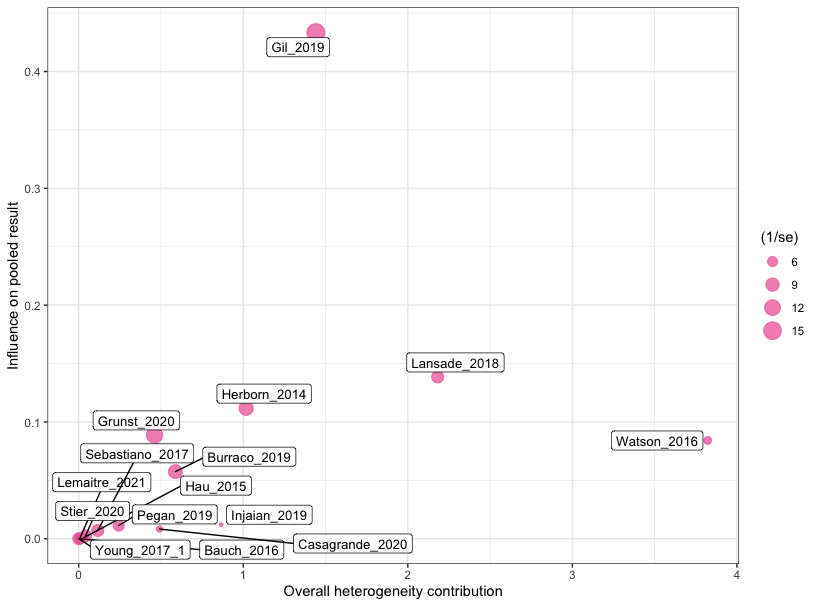

Supplement: S2 Fig — Studies can have an unequal influence on the pooled effect size and contribute to the heterogeneity of effect sizes. The horizontal axis represents Cochrane’s Q and influence on the pooled effect size on the vertical axis. (TIF) [file pone.0257370.s003.tif]

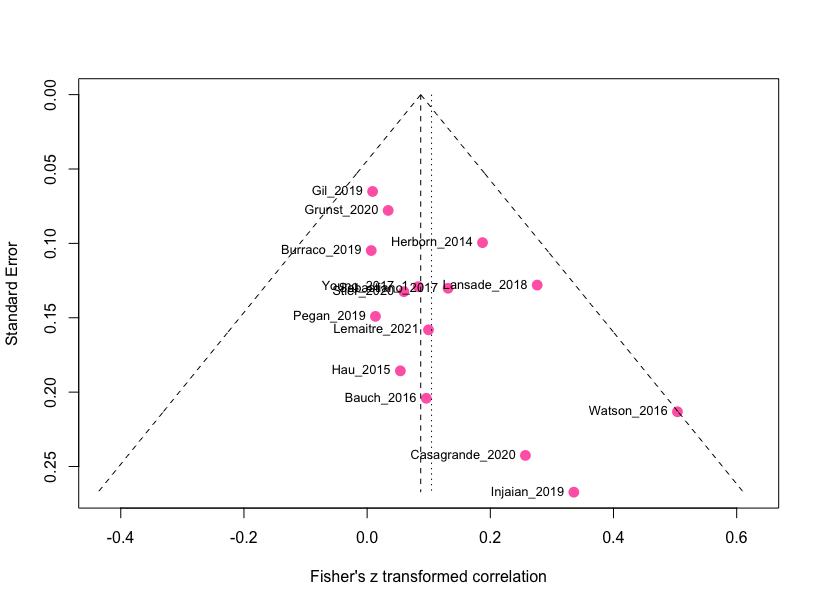

Supplement: S3 Fig — The lack of studies in the bottom left of the “funnel” demonstrates publication bias against studies with small sample sizes and small effect sizes. (TIF) [file pone.0257370.s004.tif]
